# Synthesis of titanium nitride for self-aligned gate AlGaN/GaN heterostructure field-effect transistors

**DOI:** 10.1186/1556-276X-9-590

**Published:** 2014-10-28

**Authors:** Liuan Li, Ryosuke Nakamura, Qingpeng Wang, Ying Jiang, Jin-Ping Ao

**Affiliations:** 1 Institute of Technology and Science, The University of Tokushima, Tokushima 770-8506, Japan

**Keywords:** Titanium nitride, Self-aligned gate, AlGaN/GaN heterostructure field-effect transistors, 73.40.Kp, 77.84.Bw, 73.40.Qv

## Abstract

In this study, titanium nitride (TiN) is synthesized using reactive sputtering for a self-aligned gate process. The Schottky barrier height of the TiN on n-GaN is around 0.5 to 0.6 eV and remains virtually constant with varying nitrogen ratios. As compared with the conventional Ni electrode, the TiN electrode presents a lower turn-on voltage, while its reverse leakage current is comparable with that of Ni. The results of annealing evaluation at different temperatures and duration times show that the TiN/W/Au gate stack can withstand the ohmic annealing process at 800°C for 1 or 3 min. Finally, the self-aligned TiN-gated AlGaN/GaN heterostructure field-effect transistors are obtained with good pinch-off characteristics.

## Background

The AlGaN/GaN heterostructure field-effect transistors (HFETs) are excellent candidates for high-power and high-frequency electronic devices [1,2]. To achieve a high-temperature performance, it is very desirable to produce a gate contact with a large Schottky barrier height (SBH) and an excellent thermal stability. In high-frequency applications, a self-aligned gate (SAG) process is proposed to minimize the source-to-gate and drain-to-gate distances for smaller access resistance, in which a T-shaped Schottky gate is fabricated first and then used as a mask directly for ohmic metal evaporation. Then, the Schottky gate and the ohmic electrodes are annealed simultaneously to obtain ohmic contacts [3]. An important technology to form the SAG structure is the Schottky gate which can withstand the ohmic annealing process, because the optimized ohmic contact annealing temperature of the Ti-based multilayers on GaN-based materials is usually around 800°C to 850°C [4,5]. Therefore, the Schottky gate must be able to withstand such a high temperature during the source-drain ohmic contact annealing process.

In previous studies, we have evaluated the electrical performance of Schottky contacts produced using different kinds of refractory metal nitrides such as titanium nitride (TiN), MoN, TaN, MoSiN, WTiN, ZrN, and HfN on GaN by reactive sputtering in an ambient of Ar and N_2_ mixture sputtering gas [6-8]. Considering the adhesion on GaN, sheet resistivity, reverse leakage current, SBH, and thermal stability of these devices, we regard TiN as the suitable material for the Schottky electrode. It can be obtained easily by reactive sputtering with nitrogen as the reactive gas and shows a relatively smaller resistivity, good adhesion, and less leakage current on the GaN Schottky contact.

Herein, we obtain titanium nitride (TiN) by reactive sputtering using different N_2_/Ar sputtering gas ratios. The annealing evaluation results demonstrate that the TiN/W/Au gate on AlGaN/GaN HFETs can withstand the 800°C annealing temperature for 1 or 3 min. Finally, the TiN-gated AlGaN/GaN HFETs fabricated with a self-aligned process are obtained.

## Methods

To evaluate the effect of the precursor composition, we deposited TiN films on Si-doped n-GaN (3 × 10^17^ cm^−3^ dopant density, 1-μm thick) using different N_2_/Ar sputtering gas ratios (nitrogen percentage) of 0:18 sccm (0%), 1:17 sccm (5%), 3:15 sccm (15%), 7:11 sccm (40%), 11:7 sccm (60%), and 15:3 sccm (85%). To maintain a uniform current flow between the ohmic contact and the Schottky contact, a circular Schottky pattern with a diameter of 166 μm was adopted. The ohmic contact was placed on the same side as the Schottky contact with a separating distance of 15 μm to simplify the process. A standard lift-off technology was used to form both the ohmic and the Schottky contacts. Ohmic contact was formed using a Ti/Al/Ti/Au (50/200/40/40 nm) multilayer structure, a fixed structure which is being adopted in our process system, and annealed at 800°C for 1 min. Prior to the TiN deposition process, the sample surfaces were cleaned by O_2_ plasma ashing and immersion in a diluted HCl (HCl:H_2_O = 1:1) solution for 5 min to remove any oxide layer that developed after the lithography process. The direct current (DC) sputtering power was fixed at 75 W with a chamber pressure of 0.14 Pa during the reactive sputtering.

To fabricate the TiN-gated devices by gate-first process, we firstly optimized the T-gate fabrication process on a silicon substrate using a three-layer e-beam resist technique [9]. Then, the self-aligned gate HFETs were obtained on AlGaN/GaN wafer with the optimized conditions. For the three-layer e-beam resist technique, the bottom resist layer was a ZEP520A (500 nm) layer, followed by a LOR 5B (800 nm) resist. The upper layer was also a ZEP520A (500 nm) layer. At first, the upper layer was exposed by the electron beam and developed. Then, the LOR layer was developed using a specific developer without exposure. Finally, the bottom layer was exposed by the electron beam and developed again. The stack structure of TiN/W/Au (200/50/200 nm) was then deposited to form a T-gate electrode. The drain and source regions, including the gate and the access regions, were exposed and developed using lithography technology. Ti/Al/Ti/Au multilayers with thickness of 30/120/40/40 nm were formed by lift-off technology for the drain and source contact, where the T-shaped Schottky gate was used as the mask directly. The Schottky gate and the ohmic electrodes were annealed simultaneously at 800°C for 1 min to obtain ohmic contacts.

## Results and discussion

**Figure 1:**
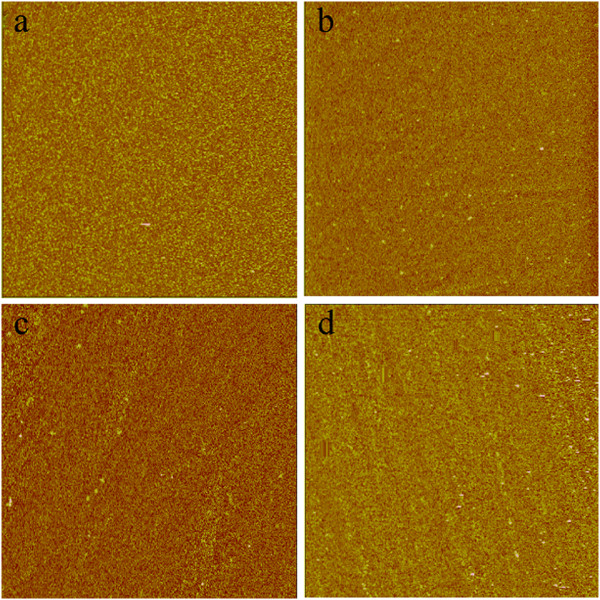
**AFM images of the TiN films deposited with various nitrogen percentages. (a)** 0%, **(b)** 15%, **(c)** 40%, and **(d)** 85%.

The typical atomic force microscope (AFM) images of the TiN samples were recorded in tapping mode in an area of 20 × 20 μm^2^ (Figure 1). Amorphous surface morphologies are represented in all the samples. The root-mean-square surface roughness are about 2.29,1.73, 0.38, 0.48, 0.53, and 0.71 nm for the films with nitrogen percentage of 0%, 5%, 15%, 40%, 60%, and 85%, respectively, which indicates that the films deposited with a mediate nitrogen content show a relatively smoother surface. The actual nitrogen contents of the samples determined from the X-ray photoelectron spectroscopy (XPS) data are 80.49%, 84.50%, 87.20%, 85.62%, and 82.06% for the samples with nitrogen deposition gas percentages of 5%, 15%, 40%, 60%, and 85%, respectively. These results show that all of the TiN films are Ti-rich with similar actual nitrogen contents, except that the sample grown with 5% N_2_ has a slightly lower actual nitrogen content than the rest.

**Figure 2:**
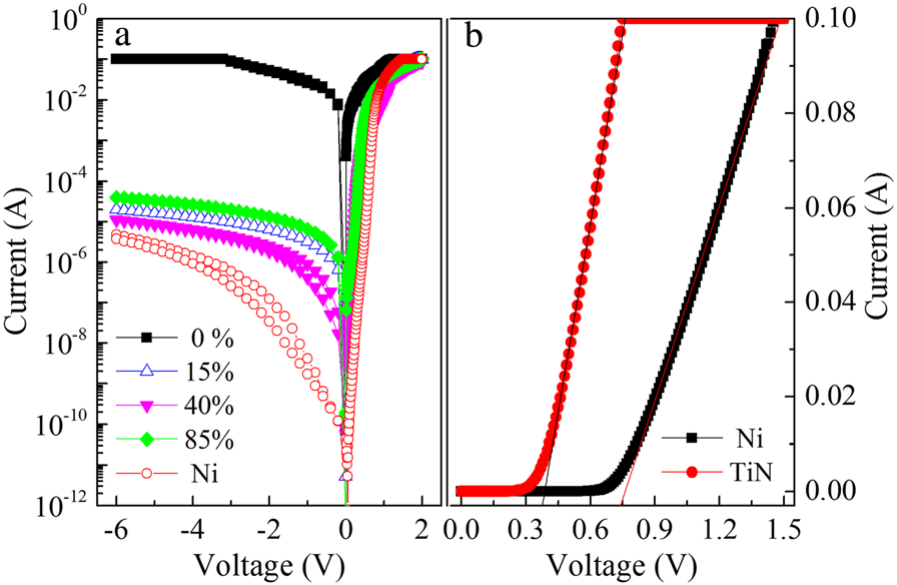
***I*-*V *characteristics and linear plot.** The *I*-*V* characteristics of the TiN Schottky diodes deposited with different N_2_/Ar ratios **(a)** and linear plot in the forward region **(b)**.

The current-voltage (*I*-*V*) characteristics of the sample with a pure Ti contact show an ohmic-like contact with a reverse leakage current quite similar to the forward current. The *I*-*V* curves of the other TiN contact samples are comparable to each other, showing good rectification characteristics and a reverse leakage current of approximately 10^−5^ A (Figure 2a). The average SBH calculated from the thermionic emission theory for the samples created with different N_2_/Ar sputtering gas ratio is around 0.5 to 0.6 eV, with ideality factors that range from 1.05 ~ 1.4 [10]. Examination of the AFM and XPS implies that the possession of similar film structure and composition causes the work function and SBH of the TiN to be essentially independent of the N_2_/Ar sputtering gas ratio [10].

For comparison, the forward region of the *I*-*V* characteristics of the Schottky diodes with Ni and TiN contacts are plotted linearly in Figure 2b. A 10-nm TiN film was formed by DC reactive sputtering in N_2_/Ar (3:15 sccm) mixed gas atmosphere with an average sputtering rate of about 0.6 nm/min. A Ni/Au (10/10 nm) electrode with an average sputtering rate of about 1.0 nm/min was prepared by radiofrequency (RF) sputtering in Ar (30 sccm) gas atmosphere under a pressure of 0.14 Pa and a power of 10 W. The SBH (ideality factor) of the TiN and Ni diode is around 0.59 eV (1.07) and 0.87 eV (1.23), respectively. These results are comparable with those of previous reports as shown in Table 1[11-13]. The turn-on voltage, which is extracted by linear fitting the forward region (Figure 2b), is about 0.38 and 0.75 V for TiN and Ni electrodes, respectively. Generally, the Ni has a higher work function (5.15 eV) than TiN (about 4.7 eV). The lower SBH and turn-on voltage are attributed to the lower work function of TiN [14].

**Table 1:** Comparison of Schottky properties of GaN diodes with TiN and Ni electrodes

**Metal**	**Work function (eV)**	**SBH (eV)**	**Ideality factor**	**Reference**
TiN	4.7	0.59	1.07	This work
Ni	5.15	0.87	1.23	This work
TiN	4.7	0.67	1.04	[11]
Ni	5.15	0.88 to 0.93	1.10 to 1.20	[12,13]

Beside, the reverse current leakage of Ni is just about 1 ~ 2 orders of magnitude lower than that of TiN diode. As compared with the value calculated from the thermionic emission theory using the Schottky barrier height of 0.59 and 0.87 eV for TiN and Ni diode, respectively, the measured leakage current of Ni is much higher than the calculated one, while those currents are in the same level for TiN. The obvious increase of leakage current of the Ni diode is attributed to the higher interface states (as compared with TiN) existing at the Ni/GaN interface caused by the interaction, leading to a defect-assisted tunneling effect [15].

**Figure 3:**
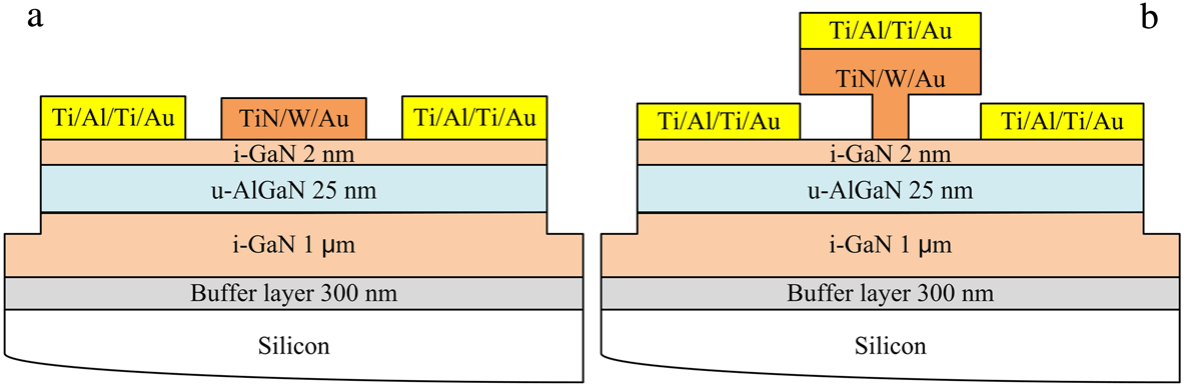
Schematics of the cross-sectional view of the conventional (a) and self-align gate (b) HFETs.

As an evaluation method for the gate-first process, we fabricated AlGaN/GaN HFETs with ohmic metals which were deposited first followed by the deposition of gate electrodes. The deposited ohmic and gate electrodes were then annealed together by changing the annealing temperature and annealing time. The schematic of the cross-sectional view of the HFETs for evaluation is shown in Figure 3a. The device fabrication started with the isolation of the mesa, which was formed by inductively coupled plasma (ICP) with an etching depth of 100 nm. The following processes were similar with the fabrication of Schottky diode mentioned above. The TiN film (about 200 nm) was formed under a N_2_/Ar sputtering gas with ratio (nitrogen percentage) of 3:15 (15%). After that, a cap layer of W/Au (30/70 nm) was deposited on the TiN layer in Ar ambient to reduce the gate resistance. The gate length and gate-source/drain spacing of the TiN/W/Au-gated AlGaN/GaN HFET were 3 and 3 μm, respectively.

**Figure 4:**
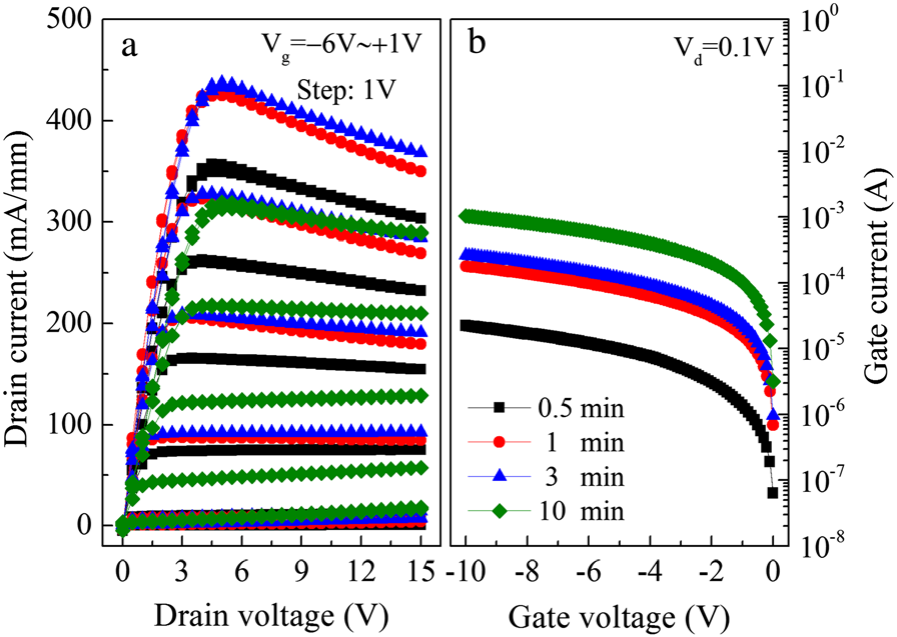
Current-voltage (a) and gate leakage current (b) characteristics of the devices annealed at different times.

It is noticed that an appropriate ohmic contact resistance of about 0.7 Ω mm can be obtained at 800°C under a mediate time of 1 or 3 min, and the sheet resistance of the gate film accreted slightly from about 0.47 to 0.67 Ω/□ when prolonging the annealing time to 10 min [16]. Figure 4a shows the *I*-*V* characteristics of HFETs for different annealing times (0.5, 1, 3, and 10 min) at 800°C. We notice that all the HFETs annealed at different times operated very well with the maximum drain current obtained for the samples annealed after 1 or 3 min (Figure 4a). Therefore, the TiN electrode can withstand the ohmic annealing temperature for 1 to 3 min. However, as shown in Figure 4b, the gate reverse leakage current increased from approximately 10^−5^ to approximately 10^−4^ A with prolong annealing. This increase in gate leakage can be contributed by the disappearance of the natural oxide on the AlGaN layer and the interface reaction between the non-nitridized Ti in the gate metal and the AlGaN layer, which can result in the increase of the defect-assisted tunneling leakage current.

**Figure 5:**
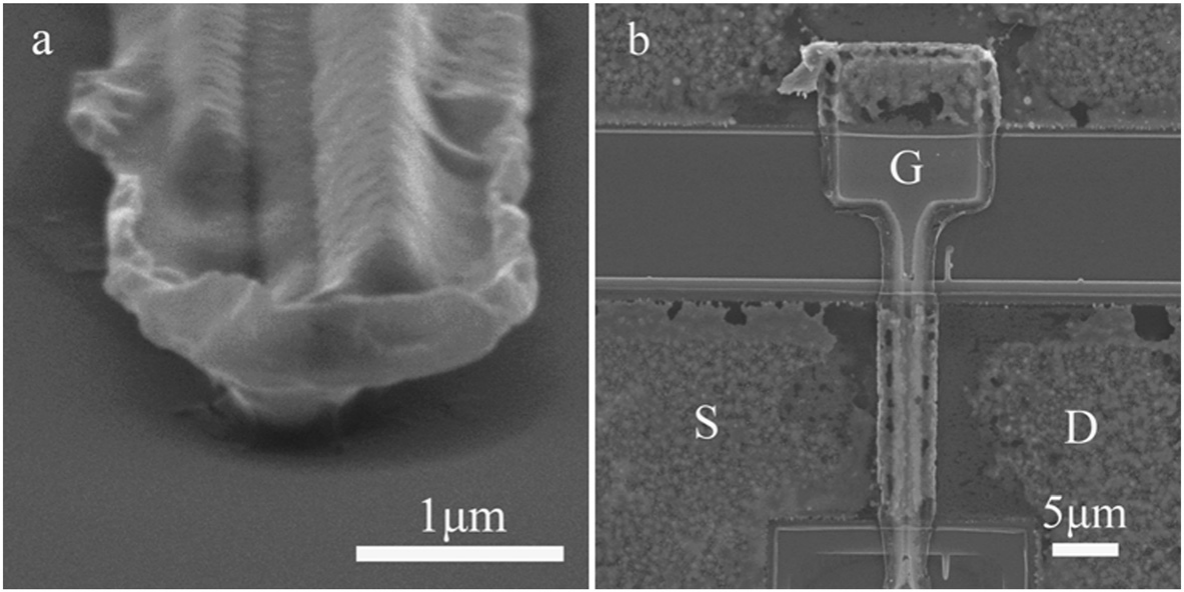
**SEM pictures of the T-gate on silicon (a) and the AlGaN/GaN HFET device (b).** S, source; G, gate; D, drain.

The cross-sectional view of the TiN-gated devices fabricated by the gate-first process is shown in Figure 3b. Figure 5a is the scanning electron micrograph (SEM) picture of the T-gate on silicon substrate, with a cross-sectional foot width of about 500 nm and a head width of about 2 μm. The AlGaN/GaN HFETs with SAG structure is also shown in Figure 5b, with a cross-sectional foot width of about 500 nm and a head width of about 4 μm. The width of the upper layer was enlarged slightly due to the changed conditions on the AlGaN/GaN HFETs substrate.

**Figure 6:**
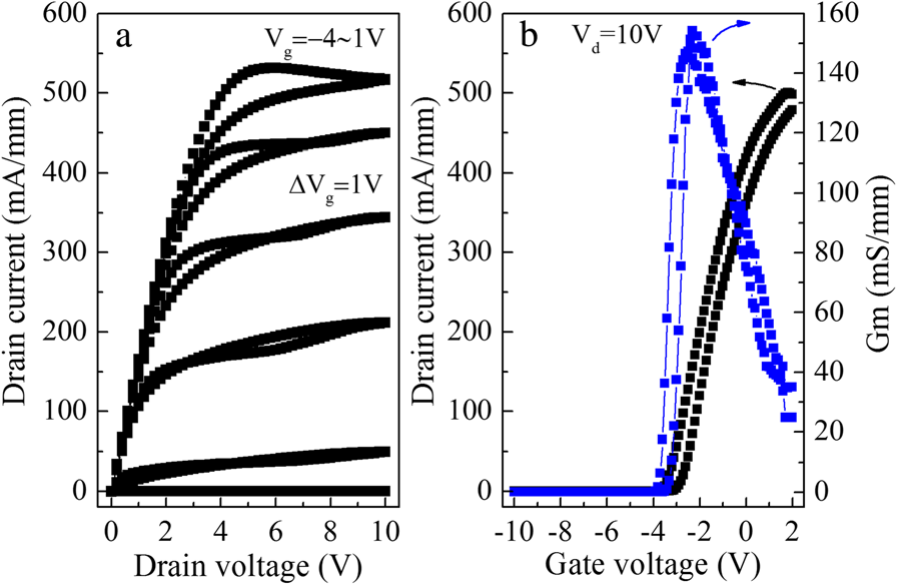
**Current-voltage characteristics (a) and transfer characteristics (b) of the self-aligned gated AlGaN/GaN HFETs.** The blue line is the Gm, and the black one is the drain current.

The *I*-*V* characteristics of HFETs show that the device with SAG structure can operate very well when the gate voltage is swept from −4 to 1 V, with a threshold voltage of about −4 V and a maximum drain current density of 540 mA/mm (Figure 6a). The transfer characteristic of the device (Figure 6b) shows that the transconductance is about 160 mS/mm. However, the surface of the electrode after annealing is rough due to the ball-up of the Au layer. More careful optimization is required to improve the morphology.

## Conclusions

We deposited TiN films with different N_2_/Ar sputtering gas ratios for self-aligned gate process. All of the samples showed good rectifying properties with an SBH of 0.5 to 0.6 eV. As compared with the conventional Ni electrode, it presented a lower turn-on voltage while the reverse leakage currents are comparable with Ni. The effects of annealing temperature and time on the electrical properties of TiN/W/Au-gate AlGaN/GaN HFETs have been investigated. It is demonstrated that the TiN/W/Au electrode could withstand the ohmic annealing process at 800°C for 1 or 3 min. Then, AlGaN/GaN HFETs with TiN gate were obtained with a self-aligned process, showing an excellent operation with a threshold voltage of about −4 V and a maximum drain current density of 540 mA/mm.

## Competing interests

The authors declare that they have no competing interests.

## Authors’ contributions

LL deposited TiN on GaN and evaluated its characterizations and drafted the manuscript. RN fabricated the devices with the gate-first process. JPA planned the experiments and agreed with the paper's publication. QW and YJ assisted in the data analysis. All authors read and approved the final manuscript.
